# Chemical Compounds, Antioxidant Activities, and Inhibitory Activities Against Xanthine Oxidase of the Essential Oils From the Three Varieties of Sunflower (*Helianthus annuus* L.) Receptacles

**DOI:** 10.3389/fnut.2021.737157

**Published:** 2021-11-19

**Authors:** Xin-Sheng Liu, Bo Gao, Zhan-De Dong, Zi-An Qiao, Min Yan, Wei-Wei Han, Wan-Nan Li, Lu Han

**Affiliations:** ^1^School of Life Sciences, Jilin University, Changchun, China; ^2^Key Laboratory for Molecular Enzymology and Engineering, Jilin University, Ministry of Education, Changchun, China; ^3^Key Laboratory for Evolution of Past Life and Environment in Northeast Asia, Jilin University, Ministry of Education, Changchun, China

**Keywords:** sunflower (*Helianthus annuus* L.), essential oils, response surface methodology (RSM), GC-MS, antioxidant analysis, xanthine oxidase (XO)

## Abstract

**Background/Aim:** Essential oils of sunflower receptacles (SEOs) have antibacterial and antioxidant potential. However, the differences of biological activities from the different varieties of sunflowers have not been studied till now. The purpose of this study was to compare the differences of chemical compounds, antioxidant activities, and inhibitory activities against xanthine oxidase (XO) of SEOs from the three varieties of sunflowers including LD5009, SH363, and S606.

**Methods:** SEOs were extracted by using the optimal extraction conditions selected by response surface methodology (RSM). Chemical compounds of SEOs were identified from the three varieties of sunflowers by gas chromatography-mass spectrometry (GC-MS). Antioxidant activities of SEOs were detected by 2,2′-azino-bis(3-ethylbenzothiazoline-6-sulfonic acid) (ABTS), 2,2-diphenyl-1-picrylhydrazyl (DPPH), and iron ion reduction ability. Inhibitory activities of SEOs against XO were measured by using UV spectrophotometer. XO inhibitors were selected from the main chemical compounds of SEOs by the high-throughput selections and molecular simulation docking.

**Results:** The extraction yields of SEOs from LD5009, SH363, and S606 were 0.176, 0.319, and 0.580%, respectively. A total of 101 chemical compounds of SEOs were identified from the three varieties of sunflowers. In addition, the results of inhibitory activities against XO showed that SEOs can reduce uric acid significantly. Eupatoriochromene may be the most important chemical compounds of SEOs for reducing uric acid. The results of antioxidant activities and inhibitory activities against XO showed that SEOs of LD5009 had the strongest antioxidant and XO inhibitory activities. The Pearson correlation coefficient (*r* > 0.95) showed that γ-terpinene, (E)-citral, and L-Bornyl acetate were highly correlated with the antioxidant activities and XO inhibitory ability.

**Conclusion:** SEOs had antioxidant activities and XO inhibitory ability. It would provide more scientific information for utilization and selection of varieties of sunflowers, which would increase the food quality of sunflowers and incomes of farmers.

## Introduction

Sunflower (*Helianthus annuus* L.) belongs to the family Compositae (Asteraceae), which originated in the south of America and spread to China in the seventeenth century (1); several varieties of sunflowers have been used as traditional medicines by Native Americans (2). Now, sunflowers are mainly planted in China (3), which are distributed widely in the north of China such as Inner Mongolia province, Jilin province, Liaoning province, Heilongjiang province, and Shanxi province (4). Among of them, Jilin province was the main producing area of sunflower (5). LD5009, SH363, and S606 are high yield sunflower varieties that are planted in Baicheng city of Jilin province. These three varieties have different commercial applications. LD5009 and SH363 are usually used for eating seeds, while S606 is used for pressing oil from seeds. It has been confirmed that the sunflower receptacles in Baicheng city of Jilin province had the biological functions of reducing uric acid and gout symptoms (6), which implied that sunflower receptacles in Baicheng city of Jilin province can be used as functional foods for treating hyperuricemia and gout. However, which variety of sunflowers had the higher biological activities is still unclear. So, it is difficult to select and distinguish the materials in food factory, which related to food quality and standard. So, it is very important to better understand the different biological activities from the different varieties of sunflowers.

Sunflower receptacles were usually used as pig feeds (7). Sometimes, they were discarded, which damaged the natural environment (8). So, it is very important to develop more applications of sunflower receptacles. Sunflower receptacles had many medicinal functions such as antigout (6), anti-inflammatory (9), antioxidant, and antimicrobial activities (10). In our previous research, it was proved that essential oils of sunflower receptacles (SEOs) had antioxidant and antimicrobial activities (10). The enzymatic hydrolysis products of sunflower receptacles adjusted the intestinal microorganisms and relieved the hyperuricemia by inhibiting the key proteins such as xanthine oxidase (XO), adenosine deaminase, and uric acid transporter 1 (11).

Sunflower oils are rich in unsaturated fatty acids such as oleic and linoleic acids (ω-6), which were considered that essential oils are good for human health (12, 13). Essential oils can be obtained by hydrodistillation (14). However, essential oils were difficult to be extracted with low extraction yield in many plants (15). Many factors of extraction would affect the extraction yields of essential oils such as liquid–solid ratio (16) and distillation time (17). Response surface methodology (RSM) can be used to select the optimal extraction conditions by evaluating the multiple parameters with less numbers of experiments (18). In previous studies, RSM was used to optimize the extraction conditions of essential oils (19) and flavonoids (20) successfully.

Different varieties of plants have different biological activities, which may be due to the different contents of chemical components. Varieties of pitaya fruits with different color peels had different antioxidant activities, which were because of the different contents of alkaloids and polyphenols (21). Different varieties of *Polygonum multiflorum* had different anti-inflammatory and antioxidant activities, which was because of the different contents of ellagic acid and quercetin (22). However, the biological activities and chemical compounds of SEOs from different varieties of sunflowers have not been reported till now.

The purpose of this study was to compare the differences of the chemical compounds, antioxidant activities, and inhibitory activities of SEOs against XO from varieties of sunflowers in Baicheng city of Jilin province in China. In this study, chemical compounds of SEOs were detected by gas chromatography-mass spectrometry (GC-MS). Antioxidant activities of SEOs were investigated by 2,2′-azino-bis(3-ethylbenzothiazoline-6-sulfonic acid) (ABTS), 2,2-diphenyl-1-picrylhydrazyl (DPPH), and iron ion reduction ability. Inhibitory activities of SEOs against XO were determined by both the experiments and molecular simulation docking. This study would provide more scientific information for using sunflower receptacles, which would reduce the waste of sunflower receptacles and increase the incomes of farmers.

## Materials and Methods

### Plant Materials

Three varieties of sunflowers were planted in Baicheng city of Jilin province in China (123°12′45″E, 44°52′23″N), which were planted in spring and harvested in autumn. Plant species were authenticated by Professor Shu-Wen Guan, School of Life Science, Jilin University. The samples of different varieties of sunflowers including LD5009, SH363, and S606 were collected in November 2020. The sunflower receptacles from the three varieties of sunflowers were air dried to remove water. The sunflower receptacles were powdered by grinder and passed through a 20-mesh standard sieve.

### Chemical Reagents

Tetracycline hydrochloride, miconazole nitrate, DPPH, and (±)-6-hydroxy-2,5,7,8-tetramethylchromane-2-carboxylic acid (Trolox) were purchased from the Source Leaf Biology Corporation Ltd. (Shanghai, China). Pentadecane, potassium persulfate (K_2_S_2_O_8_), ABTS, XO, and xanthine were purchased from the Dalian Meilun Biotechnology Corporation Ltd. (Dalian, China). The Iron Ion Reduction Capacity Kit was purchased from the Congyi Biology Corporation Ltd. (Shanghai, China). All the chemical reagents were of analytical grade.

### Extraction of Essential Oils

#### RSM for Optimal Extraction Conditions

RSM was widely used to extract the essential oils from plants (23). Therefore, RSM was used to optimize the extraction conditions of SEOs (16). Based on the single-factor experiments (24), the experiments with four factors and three levels were designed by the Box–Behnken (Stat-Ease Incorporation, Minneapolis, Minnesota, USA). The four factors included liquid–solid ratio (A), ultrasonic time (B), distillation time (C), and the concentration of sodium chloride (NaCl) (D). The ranges of independent variables and the levels are shown in Table 1. The test variable range was converted from −1 to 1.

**Table 1:** Factors and levels of RSM design.

**Factor**		**Level**		
		**−1**	**0**	**1**
A	Liquid-solid ratio (mL g^−1^)	10	15	20
B	Ultrasonic time (h)	0	1	2
C	Distillation time (h)	4	7	10
D	NaCl content (%)	0	5	10

#### Extraction of SEOs

SEOs were extracted from the three varieties of sunflowers (LD5009, SH363, and S606). According to the model and the verification test, the optimal extraction conditions of SEOs were determined by RSM as follows: the liquid–solid ratio was 17.5:1 (ml/g). The ultrasonic time was 1.5 h. The distillation time was 8 h. The NaCl content was 6%. Based on the above optimal extraction conditions by RSM, the conditions of SEOs extraction were finally determined as follows: 1,750 ml 6% NaCl was added to 100 g powders of sunflower receptacles with ultrasonic time for 1.5 h. SEOs were extracted by hydrodistillation in the Clevenger-type apparatus with distillation time for 8 h. Sodium sulfate (Na_2_SO_4_) was used to remove water from SEOs. The extractions of SEOs were repeated 30 times to provide enough SEOs for the analysis of biological activities. SEOs were stored at 2–4°C for later use.

#### Chemical Compounds of SEOs

Gas chromatography-mass spectrometry was widely used to identify and quantify the chemical compounds of essential oils (24). Chemical compounds of SEOs were analyzed by GC-MS with the Agilent (5975C, Agilent Technologies Corporation Ltd., Santa Clara, California, USA). SEOs of 10 μl were diluted with 10 μl n-hexane. The separation was achieved by using the HP-INNOWax Capillary Column (30 × 0.25 mm id, 0.25 μm film thickness) (Agilent Technologies, Santa Clara, California, USA). Helium was used as the carrier gas at a flow rate of 1 ml/min. Injector and detector temperatures were set at 250 and 280°C, respectively. The oven was maintained at 60°C for 3 min, 240°C for 5 min, and held at 240°C for 15 min. Electronic ion mode was set at 70 eV. Mass spectra were recorded in the range of m/z 50–550 amu and the ion source temperature was 230°C.

Retention indices (RIs) of the separated compounds on the HP-INNOWax Capillary Column were determined on the basis of homologous series of n-alkanes (C_9_-C_30_). The chemical compounds of SEOs were identified on the basis of comparison of their RIs. Mass spectra with published data and computer matching with the National Institute of Standards and Technology (NIST, 15.0). The library provided computer analysis of GC-MS system (25). The relative proportions of SEOs constituents were expressed as percentages obtained by peak area normalization. All the relative response factors were set as 1.

### Determination of Antioxidant Activities

Antioxidant activities of SEOs were detected by ABTS, DPPH, and the iron ion reduction. It was because that a single antioxidant method cannot accurately evaluate the antioxidant activities of SEOs.

#### Determination of ABTS Radical Scavenging Activity

ABTS radical scavenging activity was determined by the modified protocol from Kang and Song (26). ABTS working solution was prepared with 2.6 mM K_2_S_2_O_8_ and 7.4 mM ABTS, which was incubated at room temperature in the dark for 12 h and diluted with 40–45 times ethanol. We added 0.5 ml SEOs to 2 ml ABTS working solution and incubated at room temperature in the dark for 6 min. The absorbance was measured at 734 nm by a spectrophotometer (SP-752, Shanghai Instrument Corporation, Ltd., Shanghai, China). Trolox was used as a positive control.

The ABTS scavenging rate was determined by the following formula:


$$\begin{array}{l} \text{ABTS scavenging rate}\left(\text{\%}\right)=\left[\frac{\left(\text{A}0\;-\text{ A}1\right)}{\text{A}0}\right]\times 100\text{\%} \end{array}$$

where, A0 was the absorbance of the negative control without SEOs and A1 was the absorbance of the test sample with SEOs.

#### Determination of DPPH Free-Radical Scavenging Activity

DPPH free-radical scavenging activity was determined according to the modified protocol from Das et al. (27). Ethanol and DPPH were mixed to prepare 0.08 mM DPPH solution, which was stored in the dark. Here, 1 ml sample and 3 ml DPPH solution were mixed and kept at room temperature for 30 min in the dark. The absorption value was measured at 517 nm. Anhydrous ethanol and Trolox were used as negative control and positive control, respectively.

The DPPH radical scavenging capacity was determined by the following formula:


$$\begin{array}{l} \text{DPPH scavenging rate}\left(\text{\%}\right)=\left[\frac{\left(\text{A}0\;-\text{ A}1\right)}{\text{A}0}\right]\times 100\text{\%} \end{array}$$

where, A0 was the absorbance of the negative control without SEOs and A1 was the absorbance of the test sample with SEOs.

#### Determination of Iron Ion Reduction Ability

The Iron Ion Reduction Capacity Kit (A003-96T Iron Ion Reduction Ability Test Kit, Congyi Biology Corporation, Ltd., Shanghai, China) was used to determine the iron ion reducing ability of SEOs (10). Antioxidant substances can convert ferric iron of potassium ferricyanide into ferrous ions to form Prussian blue, which has a maximum absorption peak at 700 nm. The greater absorbance value, the better antioxidant activities of the SEOs.

### Inhibitory Activities Against XO

#### Determination of Inhibitory Activities of SEOs Against XO

Xanthine oxidase reacted with xanthine to produce uric acid and the content of uric acid was measured by UV spectrophotometer at 290 nm to evaluate the inhibitory activities of SEOs against XO (28, 29). The experiment of inhibitory activities of SEOs against XO was modified according to the method of Bou-Salah et al. (30).

Inhibitory activity of SEOs against XO was determined by the following formula:


$$\begin{array}{l} \text{Inhibition }\left(\text{\%}\right)=\left[\frac{\left(\text{A}-\text{B}\right)-\left(\text{C}-\text{D}\right)}{\text{A}-\text{B}}\right]\times 100\text{\%} \end{array}$$

A is the absorbance of the solution with 0.5 ml XO, 3.5 ml phosphate-buffered saline (PBS) (70 mM, pH = 7.5), and 1 ml xanthine. B is the absorbance of the solution with 4 ml PBS (70 mM, pH = 7.5) and 1 ml xanthine. C is the absorbance of the solution with 0.5 ml XO and 0.5 ml essential oils (different density), 1 ml xanthine, and 3 ml PBS (70 mM, pH = 7.5). D is the absorbance of the solution with 3.5 ml PBS (70 mM, pH = 7.5), 0.5 ml SEOs with different densities, and 1 ml xanthine.

#### Molecular Docking

High-throughput virtualization was used to screen the protease inhibitors. Chemical compounds with similar structures to allopurinol were screened from 101 chemical compounds of SEOs. Open Babel was used to process the ligand structure data file (SDF) structure information in batches and convert them into three-dimensional (3D) program database (PDB) files, which were suitable for molecular docking (31). The PDB structure of XO was obtained from the PDB database (PDBID: 3 nvw).

The AutoDock Vina Software (Scripps Research Institute, San Diego, California, USA) was used for molecular docking between the target inhibitor and XO (32), which was downloaded from the website (http://vina.scrip.edu). The results were analyzed by the Discovery Studio version 3.5 (33). The Lamarckian genetic algorithm (GA) was used to calculate the possible conformations of protein receptors and inhibitors (34).

### Statistical Analysis

All the experiments were conducted with the three replications. The one-way ANOVA and the mean comparisons were performed on antioxidant data by using the program Statistical Package for the Social Sciences (SPSS) version 20.0 (IBM Corporation, New York, USA). The Duncan's multiple range tests were used to calculate the mean values. Differences between mean values at *p* < 0.05 were considered as statistically significant. The chemical compounds, antioxidant activities, and XO inhibitory activity of SEOs were analyzed by the Pearson's correlation coefficient with R programming language (R-4.1.0 for Windows 32/64 bit).

## Results and Discussion

### Extraction of SEOs

#### RSM for Optimal Extraction Conditions of SEOs

The extraction yields of SEOs with different conditions are shown in Table 2. A second-order polynomial equation was used to express the extraction yields of SEOs as a function of the coding variables. The empirical equation was used to calculate and predict the extraction yield of sunflower receptacles. It was given as follows:


$$\begin{array}{l} \text{Extraction yield}\left(\text{\%}\right)=0.42+0.029\text{A}\\ +0.018\text{B}+0.017\text{C}+0.021\text{D}+\;0.012\text{BC}\\ -0.014\text{CD}-0.024{\text{A}}^{2}-0.018{\text{B}}^{2}-0.027{\text{C}}^{2}-0.024{\text{D}}^{2} \end{array}$$

where, A: liquid–solid ratio, B: ultrasonic time, C: distillation time, and D: NaCl content.

**Table 2:** Extraction yields of SEOs with different extraction conditions by RSM.

**Run**	**Liquid-solid**	**Ultrasonic**	**Distillation**	**NaCl**	**Extraction**
**time**	**ratio**	**time**	**time**	**content**	**yield (%)**
	**A (mL g^**−1**^)**	**B (h)**	**C (h)**	**D (%)**	
1	10.00	1.00	7.00	0.00	0.316
2	15.00	1.00	10.00	10.00	0.405
3	15.00	1.00	10.00	0.00	0.391
4	10.00	0.00	7.00	5.00	0.325
5	15.00	2.00	4.00	5.00	0.361
6	10.00	1.00	10.00	5.00	0.352
7	20.00	1.00	10.00	5.00	0.403
8	15.00	0.00	4.00	5.00	0.352
9	15.00	1.00	7.00	5.00	0.406
10	20.00	1.00	7.00	0.00	0.392
11	15.00	0.00	7.00	10.00	0.397
12	20.00	0.00	7.00	5.00	0.395
13	15.00	1.00	4.00	10.00	0.384
14	15.00	0.00	10.00	5.00	0.359
15	15.00	2.00	7.00	10.00	0.410
16	15.00	1.00	4.00	0.00	0.315
17	20.00	1.00	4.00	5.00	0.391
18	15.00	1.00	7.00	5.00	0.429
19	15.00	2.00	10.00	5.00	0.416
20	15.00	1.00	7.00	5.00	0.457
21	10.00	1.00	7.00	10.00	0.364
22	15.00	2.00	7.00	0.00	0.373
23	20.00	2.00	7.00	5.00	0.424
24	10.00	2.00	7.00	5.00	0.387
25	15.00	1.00	7.00	5.00	0.406
26	20.00	1.00	7.00	10.00	0.406
27	10.00	1.00	4.00	5.00	0.323
28	15.00	1.00	7.00	5.00	0.403
29	15.00	0.00	7.00	0.00	0.322

The ANOVA is shown in Table 3. The model was highly significant (*p* < 0.001), indicating that the equation was sufficient to predict production under any combination of variable values. A, D, and C^2^ were very important model items because the *p* < 0.001. B, C, A^2^, B^2^, and D^2^ were very important model terms because the *p* < 0.01. However, AB, AC, AD, BC, BD, and CD were not very important model terms because the *p*-value was bigger than 0.05.

**Table 3:** The ANOVA for fitted quadratic polynomial regression model.

**Variables**	**Sum of squares**	**Df**	**Mean square**	***F*-Value**	***P*-Value**
Model	0.034	14	2.460E-003	10.57	<0.0001^***^
A	9.861E-003	1	9.861E-003	42.37	<0.0001^***^
B	3.333E-003	1	3.333E-003	14.32	0.0020^**^
C	3.333E-003	1	3.333E-003	14.32	0.0020^**^
D	5.504E-003	1	5.504E-003	23.65	0.0003^***^
AB	2.723E-004	1	2.723E-004	1.17	0.2977
AC	7.225E-005	1	7.225E-005	0.31	0.5862
AD	2.890E-004	1	2.890E-004	1.24	0.2839
BC	5.760E-004	1	5.760E-004	2.47	0.1380
BD	3.610E-004	1	3.610E-004	1.55	0.2334
CD	7.562E-004	1	7.562E-004	3.25	0.0930
A^2^	3.677E-003	1	3.677E-003	15.80	0.0014^**^
B^2^	2.204E-003	1	2.204E-003	9.47	0.0082^**^
C^2^	4.749E-003	1	4.749E-003	20.40	0.0005^***^
D^2^	3.794E-003	1	3.794E-003	16.30	0.0012^***^
Residual	3.259E-003	14	2.328E-004		
Lack of fit	1.128E-003	10	1.128E-004	0.21	0.9788
Pure error	2.131E-003	4	5.327E-004		
*R^2^*	0.9136				
*R^2^* (adj)	0.8272				
*R^2^* (pred)	0.7394				
C.V. %	4.000				
Adep precision	9.858				
Cor total	0.038	28			

*Df means degree of freedom*.

** *means very significant at p < 0.01*.

*** *means highly significant at p < 0.001. A means liquid–solid ratio, B means ultrasonic time, C means distillation time, and D means the sodium chloride (NaCl) content*.

The correlation coefficient between the experimental data and the model was very high (*R*^2^ = 0.9136), indicating that the model cannot explain 8.64% of the total changes. After adjusting the *R*^2^ (pred) of 0.7394 and *R*^2^ (adj) of 0.8272, the value of the coefficient *R*^2^ (adj) was within a reasonable range (35).

In this model, the *p*-value of the liquid–solid ratio (A) was <0.001, indicating that the liquid–solid ratio had the most influence on the extraction yield of SEOs. According to the coefficients of the regression equation, the results showed that the liquid–solid ratio (A) had the greatest effect on the extraction yield of SEOs. The concentration of NaCl (D) had the greater influence on the extraction yield of SEOs. Ultrasonic time (B) and distillation time (C) had the great influence factors on the extraction yield of SEOs. So, the order of the influence factors was as follows: A > D > B > C.

**Figure 1:**
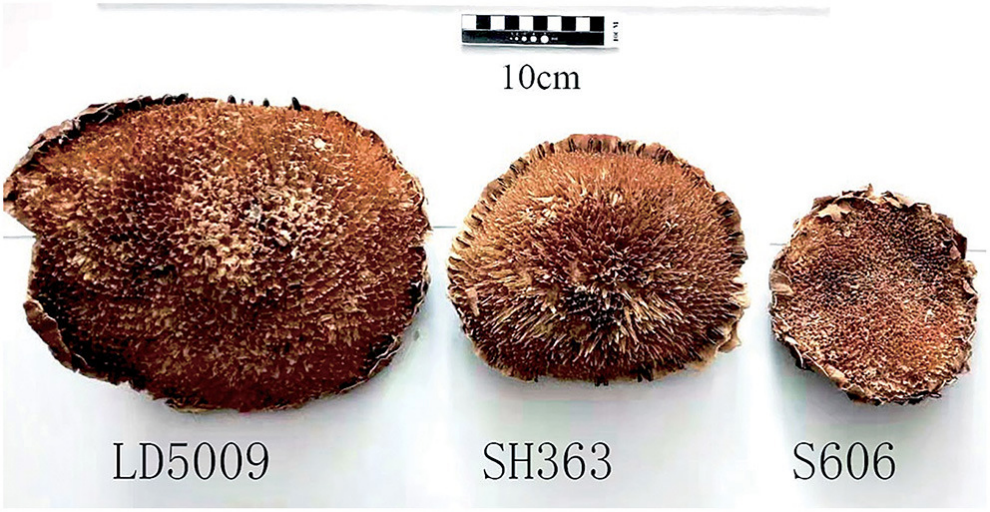
Sunflower receptacles from the three varieties of sunflowers.

**Figure 2:**
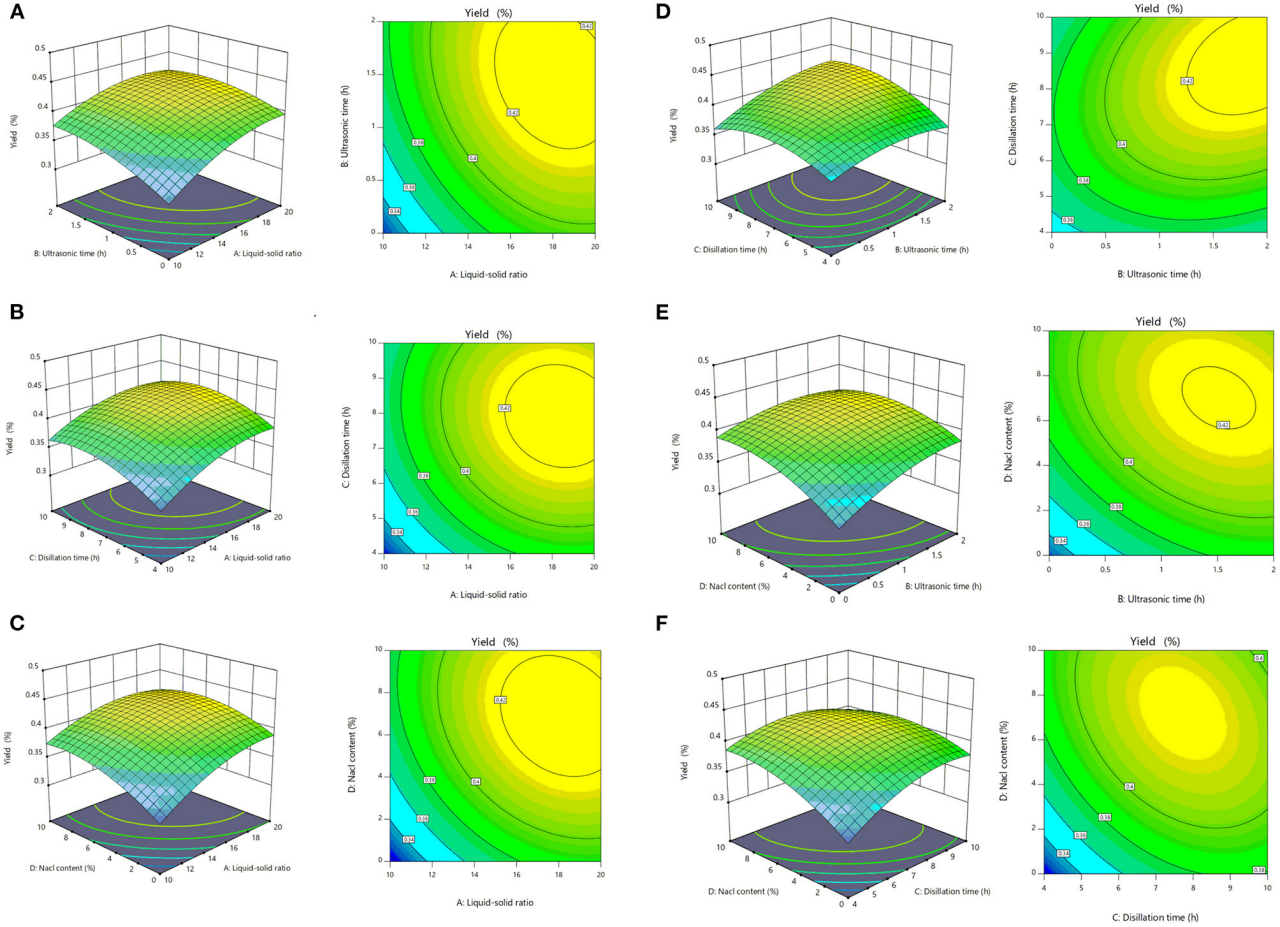
The mutual effects of the response surface plots and contour plots for the extraction yields of essential oils of sunflower receptacles (SEOs). **(A)** Ultrasound time and liquid–solid ratio, **(B)** distillation time and liquid–solid ratio, **(C)** sodium chloride (NaCl) content and liquid–solid ratio, **(D)** distillation time and ultrasonic time, **(E)** NaCl content and ultrasonic time, **(F)** NaCl content and distillation time.

The advantage of RSM was that the interaction of multiple factors can be considered at the same time on the experimental results (36). The response surface and contour lines of the interaction of A (liquid–solid ratio), B (ultrasonic time), C (distillation time), and D (NaCl content) are shown in Figure 1. The interactions among factors had a significant impact on the extraction yields of SEOs (Figure 2). The contour plots of various factors can reflect the interaction influence on the response value. Distillation time and NaCl content had the greatest influence on the extraction yields of SEOs. The contour plots of ultrasonic time and distillation time, NaCl content and ultrasonic time, and ultrasonic time and liquid–solid ratio indicated that the interactions were obvious. The results showed that the extraction yield had an upward trend. Adding an appropriate amount of inorganic salt during the extraction process could increase the extraction yield of essential oil from lavender (37).

According to the model and the verification test, the optimal extraction conditions of SEOs were determined as follows: the liquid–solid ratio was 17.5:1 (ml/g). The ultrasonic time was 1.5 h. The distillation time was 8 h. The NaCl content was 6%.

#### Analysis of the Extraction Yields of SEOs From Different Varieties of Sunflowers

The results showed that the extraction yields of SEOs from different varieties of sunflowers were much different (Table 4). The extraction yields of SEOs from LD5009, SHE363, and S606 were 0.176, 0.319, and 0.580%, respectively.

**Table 4:** The morphological characters and the extraction yields of SEOs from different varieties of sunflowers.

**Varieties**	**Diameter (cm)**	**Thickness (cm)**	**Percentage of spongy body (%)**	**Extraction yield (%)**
LD5009	25.78 ± 0.36^a^	3.09 ± 0.11^a^	54.12 ± 0.16^a^	0.176 ± 0.002^c^
SH363	19.64 ± 0.08^b^	1.87 ± 0.07^b^	38.49 ± 1.04^b^	0.319 ± 0.008^b^
S606	14.12 ± 0.19^c^	0.69 ± 0.01^c^	9.27 ± 0.10^c^	0.580 ± 0.050^a^

*Data are expressed as mean ± SD (n = 3). Values with different superscripts are significantly different (p < 0.05)*.

The extraction yield of SEOs of S606 was significantly higher than that of LD5009 and SH363, which may be related to the content of SEOs in different varieties of sunflowers. Sunflower receptacles were mainly composed of outer surrounding cells and inner spongy bodies. The morphological characters of sunflower receptacles from different varieties of sunflowers were much different (Figure 1; Table 4). The order of the sunflower receptacles size from big to small was as follows: LD5009 > SH363 > S606. The thickness and percentage of the spongy bodies of S606 were significantly lower than that of LD5009 and SH363. The results showed that the sunflower receptacles of S606 with smaller spongy bodies had much more SEOs. The sunflower receptacles of LD5009 with bigger spongy bodies had a little amount of SEOs. So, the results suggested that SEOs were not in the inner spongy bodies of sunflower receptacles, but may be in the outer surrounding cells of sunflower receptacles.

### Chemical Compounds of SEOs

**Figure 3:**
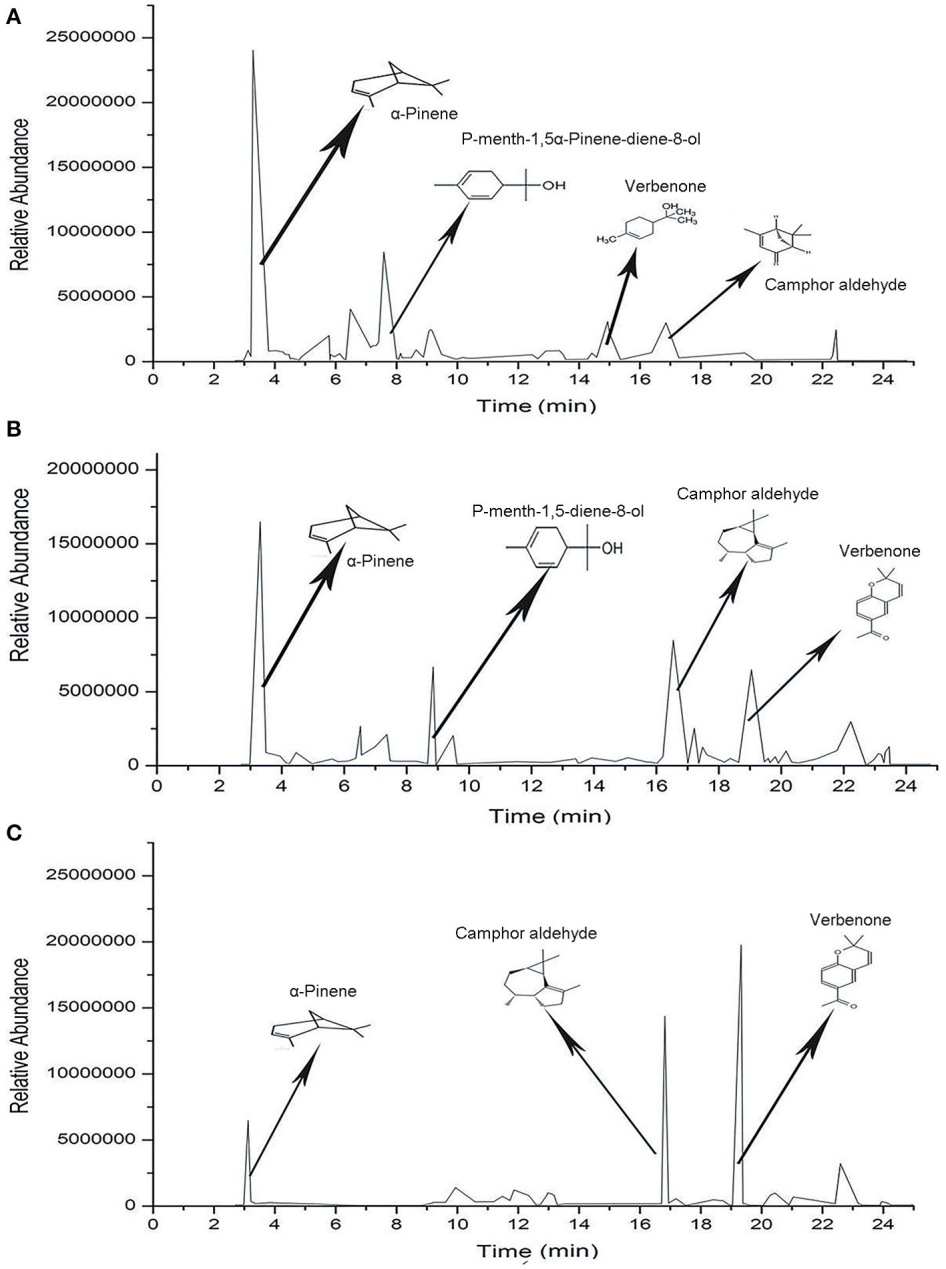
Chromatograms of the chemical compounds of SEOs from different varieties of sunflowers. **(A)** Chromatogram of chemical compounds of SEOs from LD5009. **(B)** Chromatogram of chemical compounds of SEOs from SH363. **(C)** Chromatogram of chemical compounds of SEOs from S606.

A total of 101 chemical compounds of SEOs were identified by GC-MS (Table 5; Figure 3). A total of 55 chemical compounds of SEOs were identified from LD5009 of which monoterpenoids accounted for 35.31%. Oxygenated monoterpenes accounted for 41.54%, and sesquiterpenes accounted for 4.46%. In previous study, the sunflower essential oils of “chianti,” “mammoth,” and wild-growing *H. strumosus* in north Alabama were dominated by monoterpenes, in particular α-pinene (50.65, 48.91, and 58.65%, respectively), sabinene (6.81, 17.01, and 1.91%, respectively), β-pinene (5.79, 3.27, and 4.52%, respectively), and limonene (7.2, 7.1, and 3.8%, respectively) (2). In this study, the main chemical compounds of SEOs from LD5009 were α-pinene (29.93%), p-mentha-1,5-dien-8-ol (10.54%), α-campholenal (5.52%), desmethoxyencecalin (3.86%), verbenone (3.07%), kauren-19-oic acid (3.05%), terpinene (2.92%), and α-terpineol (2.42%). A total of 61 chemical compounds of SEOs from SH363 were identified by GC-MS, accounting for 94.96% of its total contents. Among them, monoterpenoids accounted for 18.50%, oxygenated monoterpenes accounted for 33.76%, and sesquiterpenes accounted for 38.57%. The main chemical compounds of SEOs from SH363 were desmethoxyencecalin (15.46%), α-pinene (15.33%), gurjunene (11.90%), p-mentha-1,5-dien-8-ol (6.21%), campholenal (5.25%), and verbenone (6.89%). A total of 58 chemical compounds were identified from S606 by GC-MS, accounting for 91.63% of its total contents, volatile monoterpenoids accounted for 12.18%, oxygen-containing monoterpenes accounted for 13.54%, and sesquiterpenes accounted for 62.05%. The main chemical compounds of SEOs from S606 were desmethoxyencecalin (28.49%), gurjunene (19.91%), α-pinene (10.34%), dibutyl phthalate (5.12%), campholenal (2.23%), trans-verbenol (1.95%), and verbenone (1.61%). It is concluded that monoterpenes are the major compounds of essential oils in sunflower receptacles, leaves, and flowers (38).

**Table 5:** Chemical compounds of SEOs from different varieties of sunflowers.

**No**.	**Compound**	**Formula**	**RT**	**RI**	**Content (%)**		
					**LD5009**	**SH363**	**S606**
1	α-Pinene	C_10_H_16_	2.80	928	29.93	15.33	10.34
2	2,4(10)-Thujadiene	C_10_H_14_	3.22	961	1.01	–	0.56
3	β-Thujene	C_10_H_16_	3.61	1,024	1.07	0.82	0.32
4	Terpinene	C1_0_H_16_	3.67	1,032	2.92	–	–
5	Ethanone-1-(2-Methyl-1-cyclopenten-1-yl)	C_10_H_16_	3.94	1,042	0.69	–	–
6	1,8-Dehydrocineole	C_10_H_16_O	3.95	1,059	0.69	0.60	0.43
7	1,3,8-*p*-Menthatriene	C_10_H_14_	3.96	1,062	–	0.17	0.32
8	1,5,8-*p*-Menthatriene	C_10_H_1_4	4.97	1,071	0.28	–	–
9	*cis*-1,4-Dihydroxycyclooctane	C_8_H_16_O_2_	5.03	1,075	–	0.13	0.10
10	*p*-Cymene	C_10_H_1_4	5.58	1,084	–	0.83	–
11	*d*-Limonene	C_10_H_16_	6.65	1,091	0.16	–	–
12	γ-Terpinene	C_10_H_16_	7.19	1,096	0.44	0.21	–
13	(1R)-*cis*-Verbenol	C_10_H_16_O	7.64	1,098	0.13	0.13	0.10
14	1-Methyl-2-phenylcyclopropane	C_10_H_12_	8.73	1,102	0.48	0.42	0.29
15	Campholenal	C_10_H_16_O	9.74	1,118	5.52	5.25	2.23
16	1-(1-Methylethyl)-2-(2-methyl-1-methylenepropyl)-Cyclopropane	C_11_H_20_	10.85	1,123	–	0.42	–
17	1,7,7-Trimethylbicyclo[2.2.1]hept-5-en-2-ol	C_10_H_16_O	11.86	1,138	–	0.30	–
18	6-Camphenone	C_10_H_14_O	11.87	1,139	0.78	–	0.46
19	*o*-Cymene	C_10_H_14_	12.25	1,141	0.33	–	0.46
20	Benzyloxypropan	C_10_H_14_O	12.42	1,143	–	–	0.32
21	Benzene-butoxymethyl	C_11_H_16_O	12.43	1,146	–	0.67	–
22	α-Ethyl-benzeneethanol	C_10_H_14_O	12.43	1,147	0.52	–	–
23	*trans*-Sabinol	C_10_H_16_O	12.59	1,151	0.54	–	–
24	Sabinyl acetate	C_12_H_18_O_2_	12.59	1,152	1.24	1.17	1.24
25	3,3,5,5-Tetramethylcyclopentene	C_9_H_16_	12.59	1,156	–	–	0.67
26	*trans*-Verbenol	C_10_H_16_O	12.66	1,160	1.11	1.96	1.95
27	p-Mentha-1,5-dien-8-ol	C_10_H_16_O	12.73	1,163	10.54	6.21	1.27
28	3,5-Dihydroxyacetophenone	C_8_H_8_O_3_	12.75	1,165	0.31	–	0.16
29	1-(1,4-Dimethylcyclohex-3-en-1-yl)-ethanone	C_10_H_16_O	12.75	1,169	–	0.30	–
30	Isopinocamphone	C_10_H_16_O	12.93	1,170	0.22	–	–
31	Pinocarvone	C_10_H_14_O	12.97	1,172	0.81	–	–
32	(*e*)-Citral	C_10_H_16_O	13.16	1,176	0.38	0.28	0.19
33	2-Prenylcyclopentanone	C_10_H_16_O	13.16	1,178	–	–	0.19
34	2-Pentyl-2-cyclopenten-1-one	C_10_H_16_O	13.17	1,179	0.38	–	–
35	α-Terpinyl formate	C_11_H_18_O_2_	13.18	1,181	–	0.12	–
37	(-)-Terpinen-4-ol	C_10_H_18_O	13.18	1,186	1.14	–	0.20
37	α-Terpineol	C_10_H_18_O	13.38	1,188	2.42	0.28	–
38	Myrtenal	C_10_H_14_O	13.50	1,189	2.93	–	1.31
39	(-)-Verbenone	C_10_H_14_O	13.69	1,190	3.07	6.89	1.61
40	*trans*-Carveol	C_10_H_16_O	13.79	1,192	2.06	1.13	1.03
41	(+)-*trans*-Chrysanthenyl acetate	C_12_H_18_O_2_	13.80	1,198	–	1.00	–
42	Norbornene	C_10_H_16_	13.85	1,407	–	0.21	–
43	5,7-Dimethylenebicyclo (2.2.2) oct-2-ene	C_10_H_12_	13.98	1,410	–	0.21	–
44	δ-Elemene	C_15_H_24_	13.99	1,414	–	–	0.18
45	L-Bornyl acetate	C_12_H_20_O_2_	13.99	1,418	0.69	0.43	0.27
46	Bornyl acetate	C_12_H_20_O_2_	14.31	1,419	–	–	0.27
47	*p*-Cymen-7-ol	C_10_H_14_O	14.80	1,423	0.23	0.14	–
48	γ-Maaliene	C_15_H_24_	14.80	1,426	–	–	0.30
49	2-Methoxy-4-vinylphenol	C_9_H_10_O_2_	14.84	1,429	–	0.48	–
50	Myrtenyl acetate	C_12_H_18_O_2_	14.99	1,430	0.41	–	–
51	1,4-*p*-Menthadien-7-ol	C_10_H_16_O	15.17	1,446	0.33	0.27	–
52	Eugenol	C_10_H_12_O_2_	15.58	1,456	0.40	0.28	0.13
53	(-)-Aristolene	C_15_H_24_	15.77	1,462	0.34	0.50	–
54	β-Patchoulene	C_15_H_24_	16.71	1,469	0.28	–	0.30
55	β-Sesquiphellandrene	C_15_H_24_	16.71	1,471	–	0.23	–
56	(+)-*b*-Cedrene	C_15_H_24_	16.75	1,475	–	0.23	–
57	Gurjunene	C_15H24_	16.82	1,476	–	11.90	19.91
58	2-Tridecanone	C_15_H_22_	16.82	1,479	1.05	2.36	0.37
59	Bicyclosesquiphellandrene	C_13_H_26_O	16.94	1,480	–	0.15	–
60	β-Bisabolene	C_15_H_24_	17.01	1,485	0.63	2.09	0.84
61	α-Muurolene	C_15_H_2_4	17.19	1,489	–	0.15	–
62	Spathulenol	C_15_H_24_O	17.50	1,497	0.24	0.45	0.25
63	*d*-Cadinene	C_15_H_24_	17.73	1,499	0.67	0.67	–
64	β-Calacorene	C_15_H_20_	17.73	1,503	0.20	0.22	–
65	Junenol	C_15_H_26_O	17.96	1,512	–	–	0.76
66	Demethoxyencecalinol	C_13_H_16_O_2_	18.41	1,521	–	–	0.64
67	*cis*-Calamenene	C_15_H_22_	18.73	1,543	–	–	0.17
68	T-Cadinol	C_15_H_26_O	18.93	1,846	–	0.24	0.15
69	α-Elemene	C_15_H_24_	19.04	1,902	0.40	0.21	0.37
70	Desmethoxyencecalin	C_13_H_14_O_2_	19.24	1,914	3.86	15.46	28.49
71	Ylangenol	C_15_H_24_O	19.33	1,916	–	–	0.33
72	9-Hydroxy-Isolongifolene	C_15_H_24_O	19.38	1,920	–	–	–
73	*cis*-Lanceol	C_15_H_24_O	19.55	1,928	–	0.19	–
74	Isospathulenol	C_15_H_24_O	19.58	1,936	–	–	0.14
75	Germacratrien-1-ol	C_15_H_24_O	19.61	1,942	0.82	0.56	0.10
76	Longifolenaldehyde	C_15_H_24_O	19.76	1,964	–	0.15	-
77	*trans*-Valerenyl acetate	C_17_H_26_O_2_	19.76	1,968	–	–	0.09
78	Eupatoriochromene	C_13_H_14_O_3_	20.05	1,970	2.74	1.35	0.92
79	Aristolone	C_15_H_22_O	20.28	1,972	–	–	1.56
80	8,9-Dehydro-neoisolongifolene	C_15_H_22_	20.45	1,973	0.20	–	0.14
81	1-Hexadecanol	C_16_H_34_O	20.90	1,974	–	0.22	–
82	*n*-Hexadecanoic acid	C_16_H_32_O_2_	20.90	1,976	0.87	2.81	1.11
83	Hexadecanoic acid,methyl ester	C_17_H_34_O_2_	21.04	1,979	–	0.16	–
84	Cyclododecanemethanol	C_13_H_26_O	21.31	1,981	–	0.43	–
85	Dihydrodehydrocostus lactone	C_15_H_20_O_2_	21.44	1,984	–	0.57	0.30
86	Dibutyl phthalate	C_16_H_22_O_4_	22.42	1,995	2.74	2.77	5.12
87	(+)-13-Epi-Manoyl oxide	C_20_H_34_O	22.59	1,997	0.50	0.37	–
88	13-Epimanoyl oxide	C_20_H_34_O	23.03	1,998	–	–	0.29
89	Linolic acid	C_18_H_32_O_2_	23.18	2,000	–	0.76	–
90	10(E),12(Z)-Conjugated linoleic acid	C_18_H_32_O_2_	23.21	2,002	0.17	0.69	–
91	1,9-Heptadecadiene-4,6-diyn-3-ol	C_17_H_24_O	23.29	2,004	0.84	–	–
92	9,12-Octadecadienoic acid (Z,Z)-methyl ester	C_19_H_34_O_2_	23.29	2,006	–	–	0.14
93	Methyl isopimarate	C_21_H_32_O_2_	23.30	2,007	0.20	0.18	–
94	24-Noroleana-3,12-diene	C_29_H_46_	23.30	2,008	–	–	0.12
95	10(E),12(Z)-Conjugated linoleic acid	C_18_H_32_O_2_	23.89	2,011	–	–	0.54
96	*cis*-13-Octadecenoic acid	C_18_H_32_O_2_	23.94	2,018	–	–	0.41
97	Isopimaric acid methyl ester	C_21_H_32_O_2_	24.11	2,028	–	–	0.15
98	20-Methyl-5-pregnene-3,20-diol	C_20_H_34_O	24.24	2,031	–	–	0.14
99	*H*-Kauran-16-ol	C_20_H_34_O	24.45	2,049	0.23	–	–
100	Kaur-16-en-18-al	C_20_H_30_O	25.23	2,092	0.60	0.85	0.17
101	Kauren-19-oic acid	C_20_H_30_O_2_	25.49	2,098	3.05	1.19	0.71
	Total				94.79	94.96	91.63

*RT means retention time. RI means retention index calculated by the retention time in relation to that of C_9_-C_30_ n-alkanes on the HP-INNOWax Capillary Column*.

### Analysis of Antioxidant Activities

**Figure 4:**
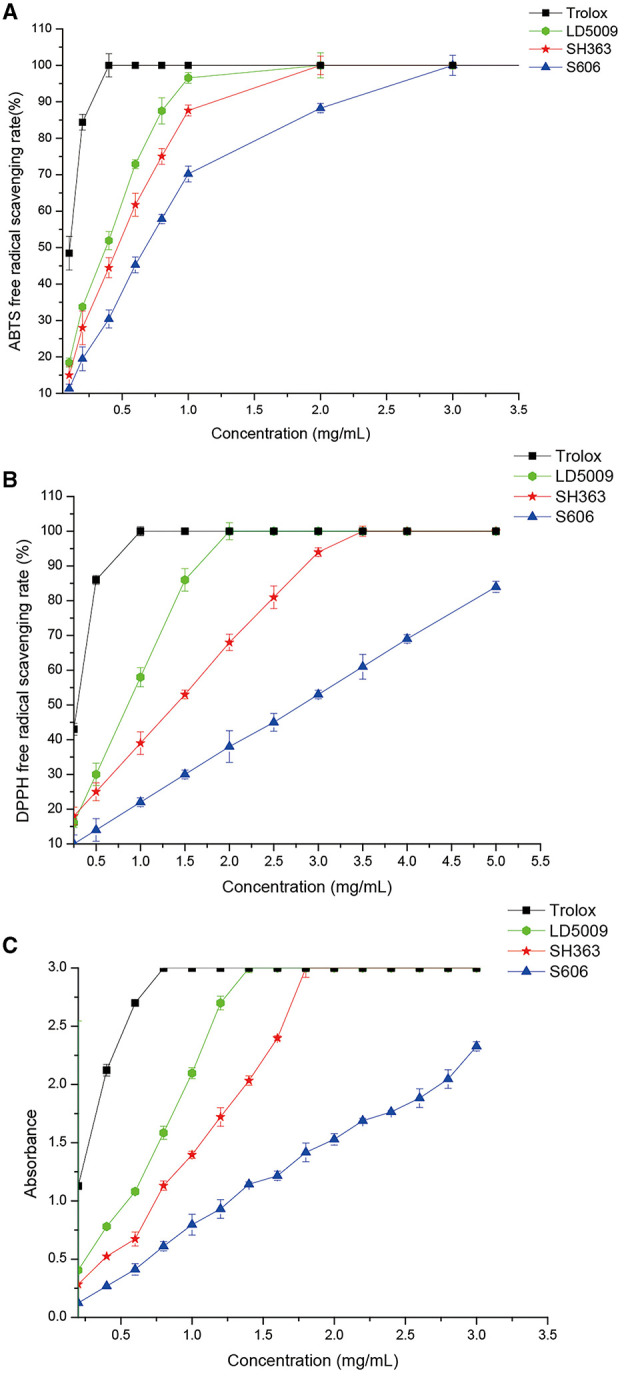
Antioxidant activities of SEOs from the different varieties of sunflowers. **(A)** ABTS free-radical scavenging rate of SEOs from different varieties of sunflowers. **(B)** DPPH free radical scavenging rate of SEOs from different varieties of sunflowers. **(C)** Iron ion reduction ability of SEOs from different varieties of sunflowers.

All the SEOs from the three varieties of sunflowers had antioxidant activities (Figure 4). However, the antioxidant activities of SEOs from LD5009, SH363, and S606 were different. The antioxidant activities of SEOs were listed as followers: LD5009 > SH363 > S606. The antioxidant activities of SEOs in the three varieties of sunflowers were increased with high SEOs concentration, which indicated that the antioxidant activities of SEOs were highly dependent on the concentrations of SEOs.

Previous studies proved that monoterpenoids (carveol) in essential oils had antioxidant activities (39, 40). Eugenol, γ-terpinene, and α-terpinolene had good antioxidant activities. However, α-pinene had very low antioxidant activity, while terpinene and α-terpinene have no antioxidant activity (40). Carveol, eugenol, γ-terpinene, α-pinene, and α-terpinolene were all belonged to monoterpenoids (Table 5). The contents of monoterpenoids of SEOs from LD5009 and SH363 were much higher than that of SEOs from S606. Antioxidant activities of SEOs from LD5009 and SH363 were higher than that of S606. So, antioxidant activities of SEOs related with the content of monoterpenoids.

### Inhibitory Activity of SEOs Against XO

#### Determination of XO Inhibitory Rate

**Figure 5:**
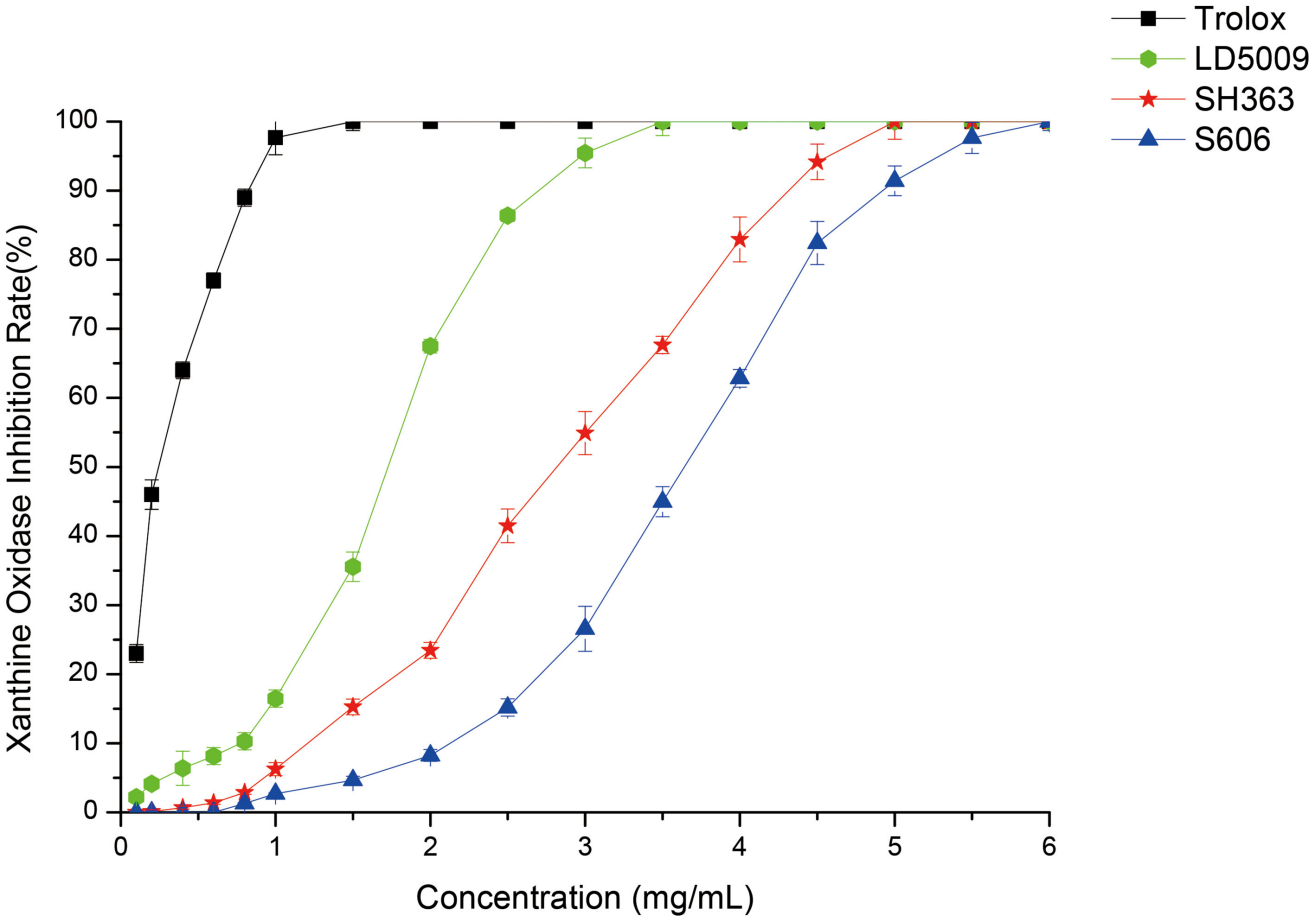
Inhibitory activities of SEOs from different varieties of sunflowers on XO.

As shown in Figure 5, SEOs had a significant inhibitory effect on XO. SEOs of different sunflower varieties had different inhibitory abilities against XO. The inhibitory activities of SEOs against XO were ordered as follows: LD5009 > SH363 > S606. Previous studies showed that gout was caused by hyperuricemia. Uric acid was produced by the reaction of XO with xanthine (41). XO inhibitors may inhibit the activity of XO and reduce the content of uric acid in the blood, thereby avoid the occurrence of hyperuricemia (42). SEOs had a good inhibitory activities against XO. SEOs had the functions for reducing uric acid and inhibiting the production of gout.

#### Molecular Simulation Docking of SEOs and XO

**Figure 6:**
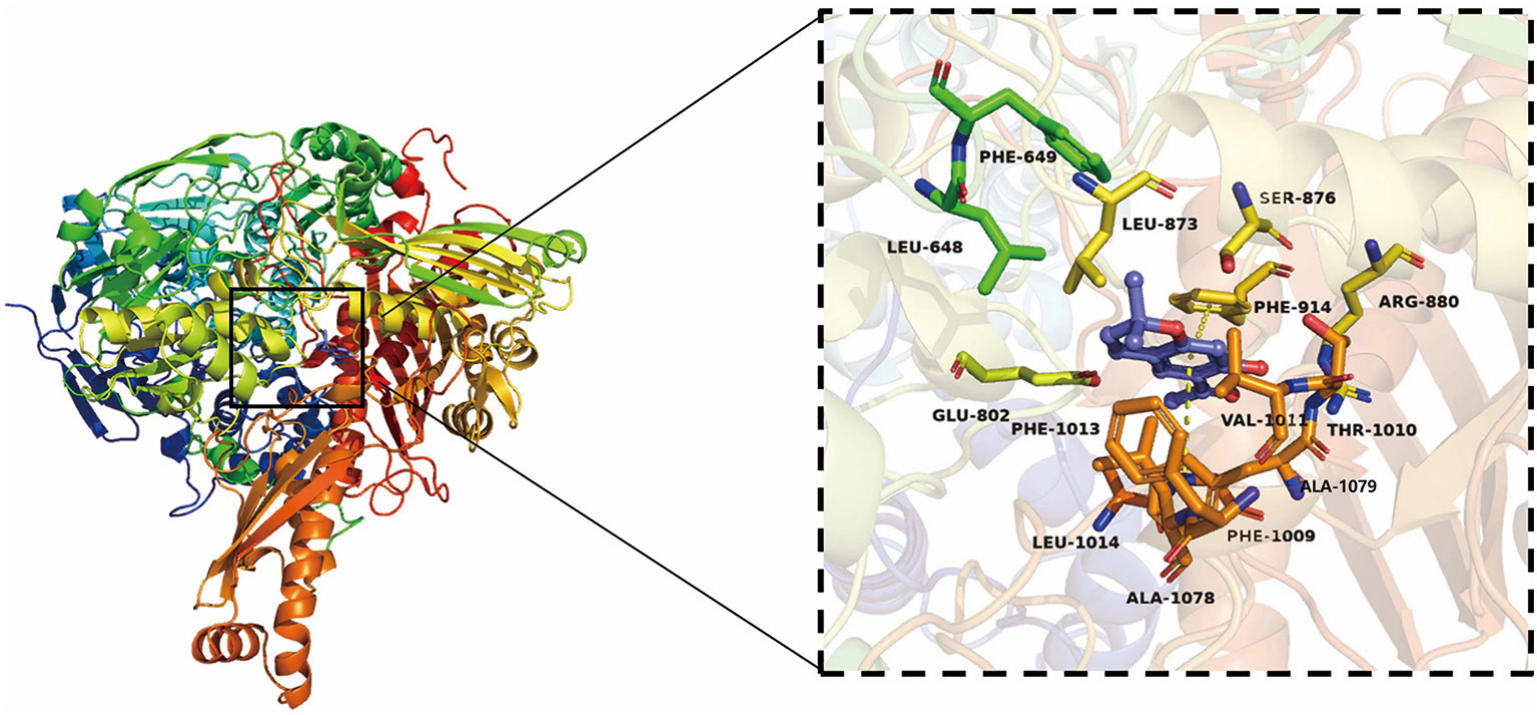
Three-dimensional (3D) structure of XO and the active residues of XO binding to eupatoriochromene.

Eupatoriochromene was screened to prove its inhibitory activities against XO from the chemical compounds of SEOs by high-throughput virtual screening and molecular simulation docking. The results of molecular simulation docking showed that eupatoriochromene can bind to XO very well (Figure 6; Supplementary Figure 1).

The results of molecular simulation docking showed that the key active residues of XO combined to eupatoriochromene were Val1011, Phe1013, Leu648, Phe649, Leu1014, and Leu873, which formed Pi-alkyl in Figure 6. The benzene ring of Phe1009, Phe914, and eupatoriochromene formed Pi-Pi conjugated double bond. Ser876, Thr1010, Arg880, Ala1079, Ala1078, Glu802, and eupatoriochromene formed van der Waals forces. These weak intermolecular interactions played an important role in the interactions between eupatoriochromene and XO. Competitive inhibitors were inserted into the hydrophobic gap of XO to occupy its catalytic activity center, hindering the entry of the substrate and leading to its inactivation (43). Eupatoriochromene was a competitive inhibitor of xanthine in the system. Therefore, the main chemical compounds of SEOs may be eupatoriochromene, which had an inhibitory effect on XO.

The inhibitory abilities of SEOs on XO were listed as follows: LD5009 > SH363 > S606. The contents of eupatoriochromene in the three SEOs were 2.74% (LD5009), 1.35% (SH363), and 0.92% (S606). So, the inhibitory ability of SEOs against XO was possibly correlated with the content of eupatoriochromene in SEOs. The positive correlation phenomenon corresponded to the results of molecular simulation docking between XO and the chemical compounds of SEOs. It could be concluded that the inhibitory ability of SEOs effect on XO may be due to the main chemical components of SEOs such as eupatoriochromene.

### Correlation Analysis Among the Chemical Compounds Antioxidant Activity and XO Inhibitory Activity

The Pearson correlation analysis is widely used in analysis of the correlation analysis between chemical compounds of SEOs and biological activities. The larger the Pearson coefficient (*r*), the greater the correlation between the chemical compounds and the biological activities (44). The results of correlation analysis showed that the chemical compounds of SEOs had the great correlation with antioxidant activities and XO inhibitory activity (Table 6; Supplementary Figure 2). The Pearson correlation coefficients of 12 chemical compounds of SEOs with antioxidant activities and XO inhibitory activity were *r* > 0.8, indicating that these chemical compounds of SEOs were highly positively correlated with antioxidant activities and XO inhibitory activity. The Pearson correlation coefficients of γ-terpinene, (E)-citral, and L-Bornyl acetate with both antioxidant activities and XO inhibition were *r* > 0.95, indicating that these chemical compounds of SEOs may play a major role in both the antioxidant activities and XO inhibitory activity. Some of the chemical compounds of SEOs were highly correlated only with the antioxidant activities (*r* > 0.95), while some of the chemical Compounds of SEOs were not highly correlated with XO inhibitory activity (*r* > 0.8), such as β-thujene, 1,8-dehydrocineole, 1-methyl-2-phenylcyclopropane, and p-mentha-1,5-dien-8-olandgermacratrien-1-ol. The results of molecular simulation docking showed that eupatoriochromene can bind to the active sites of XO and inhibit the activity of XO. Therefore, it can be concluded that eupatoriochromene might play an important role in the inhibition of XO activity.

**Table 6:** The Pearson correlation coefficient (r) of the chemical compounds of SEOs with antioxidant activity and XO inhibition ability.

**Compounds**	**ABTS**	**DPPH**	**Iron**	**XO**
α-Pinene	0.899	0.882	0.862	0.887
β-Thujene	0.999	0.999	0.981	0.815
1,8-Dehydrocineole	0.999	0.999	0.984	0.8244
γ-Terpinene	0.979	0.971	0.999	0.951
1-Methyl-2-phenylcyclopropane	0.999	0.999	0.977	0.804
p-Mentha-1,5-dien-8-ol	0.990	0.984	0.999	0.894
(e)-Citral	0.978	0.970	0.999	0.952
trans-Carveol	0.819	0.896	0.907	0.999
L-Bornyl acetate	0.951	0.959	0.99	0.958
Germacratrien-1-ol	0.999	0.998	0.987	0.833
Eupatoriochromene	0.891	0.873	0.956	0.991
Kauren-19-oic acid	0.876	0.856	0.946	0.995

*ABTS means 2,2′-azino-bis(3-ethylbenzothiazoline-6-sulfonic acid) free-radical scavenging ability. DPPH means 2,2-diphenyl-1-picrylhydrazyl free-radical scavenging ability. Iron means iron ion reduction ability. XO means XO inhibitory ability*.

### Application of SEOs

In this study, the results of antioxidant activities by ABTS, DPPH, and iron ion reduction ability showed that SEOs had antioxidant activities, implied that SEOs can be used as natural antioxidant to prevent food oxidation, which may keep food and fruit fresh for longer time. The antioxidant activities of SEOs from LD5009 were the highest among these three varieties of sunflower including LD5009, SH363, and S606. The seeds of LD5009 were always eaten directly as snacks, while the receptacles of LD5009 were discarded. It would provide a new application of sunflower receptacles in food sector that it can be developed to extract SEOs as natural antioxidant instead of throwing it away. SEOs had high XO inhibitory ability, implied that it can be used as healthcare food to reduce the uric acid for the patients with hyperuricemia (20), which would provide the evidence of its applications for hyperuricemia and gout (6).

## Conclusion

The optimal extraction conditions of SEOs were selected by RSM with liquid–solid ratio of 17.5:1 (ml/g), ultrasonic time of 1.5 h, distillation time of 8 h, and the concentration of NaCl of 6%. SEOs were extracted from three varieties of sunflowers (LD5009, SH363, and S606) by hydrodistillation in the Clevenger-type apparatus. The extraction yields of LD5009, SHE363, and S606 were 0.176, 0.319, and 0.580%, respectively. A total of 101 chemical compounds of SEOs were identified from the three varieties of sunflowers by GC-MS. The results of the antioxidant activities of SEOs showed that the antioxidant activities of SEOs from the three varieties of sunflowers were quite different (LD5009 > SH363 > S606). The analysis of the inhibitory activity of SEOs against XO and molecular simulation docking revealed that SEOs had strong inhibitory effect on XO, which may be related to eupatoriochromene of SEOs. The Pearson correlation coefficient (*r* > 0.95) showed that γ-terpinene, (E)-citral, and L-Bornyl acetate were highly correlated with antioxidant activities of SEOs and XO inhibitory ability. The results of the antioxidant activities and XO inhibitory activity both showed that SEOs have good antioxidant activities and XO inhibitory ability. This study would provide more scientific information for utilization of sunflower receptacles and good selection of different varieties of sunflowers, which would increase the incomes of farmers.

## Data Availability Statement

The original contributions presented in the study are included in the article/supplementary material, further inquiries can be directed to the corresponding author/s.

## Author Contributions

LH, BG, W-WH, and W-NL conceived and designed the study. X-SL, MY, and Z-AQ performed the experiments. X-SL and LH analyzed the data. X-SL, LH, and Z-DD wrote the manuscript. All the authors have read and agreed to the published version of the manuscript.

## Funding

This study was funded by the National Natural Science Foundation of China (Grant No. 31870201), received research funding from the Key Laboratory for Evolution of Past Life and Environment in Northeast Asia by the Ministry of Education of China, and received funding from Jilin Teyi Food Technology Corporation Ltd.

## Conflict of Interest

The authors declare that this study received funding from Jilin Teyi Food Technology Corporation Ltd. The funder had the following involvement in the study: design, sample collection and the decision to submit it for publication.

## Publisher's Note

All claims expressed in this article are solely those of the authors and do not necessarily represent those of their affiliated organizations, or those of the publisher, the editors and the reviewers. Any product that may be evaluated in this article, or claim that may be made by its manufacturer, is not guaranteed or endorsed by the publisher.
