# Supplementary material for: Chemical Compounds, Antioxidant Activities, and Inhibitory Activities Against Xanthine Oxidase of the Essential Oils From the Three Varieties of Sunflower (Helianthus annuus L.) Receptacles
Source: Front Nutr. 2021 Nov 19;8:737157. doi: 10.3389/fnut.2021.737157 (PMC8641733; doi:10.3389/fnut.2021.737157)
Supplement: Supplementary file 1 [file Image_1.pdf]

Supplementary Material

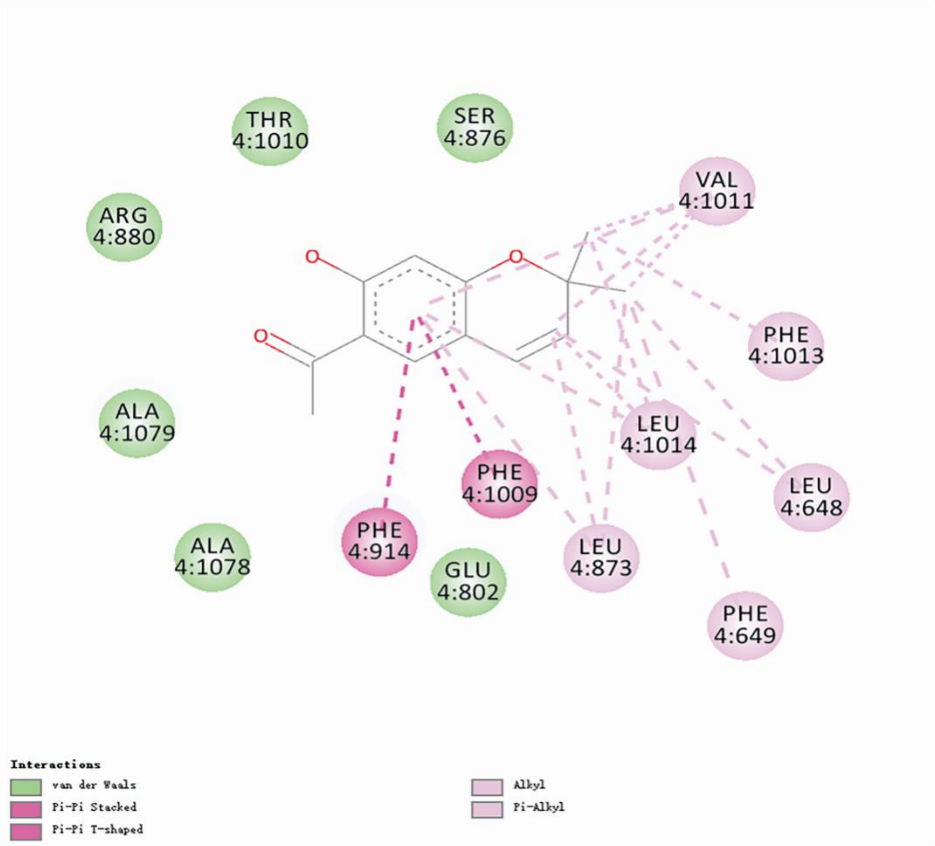

FIGURE S1 Active residues of eupatoriochromene binding to XO.

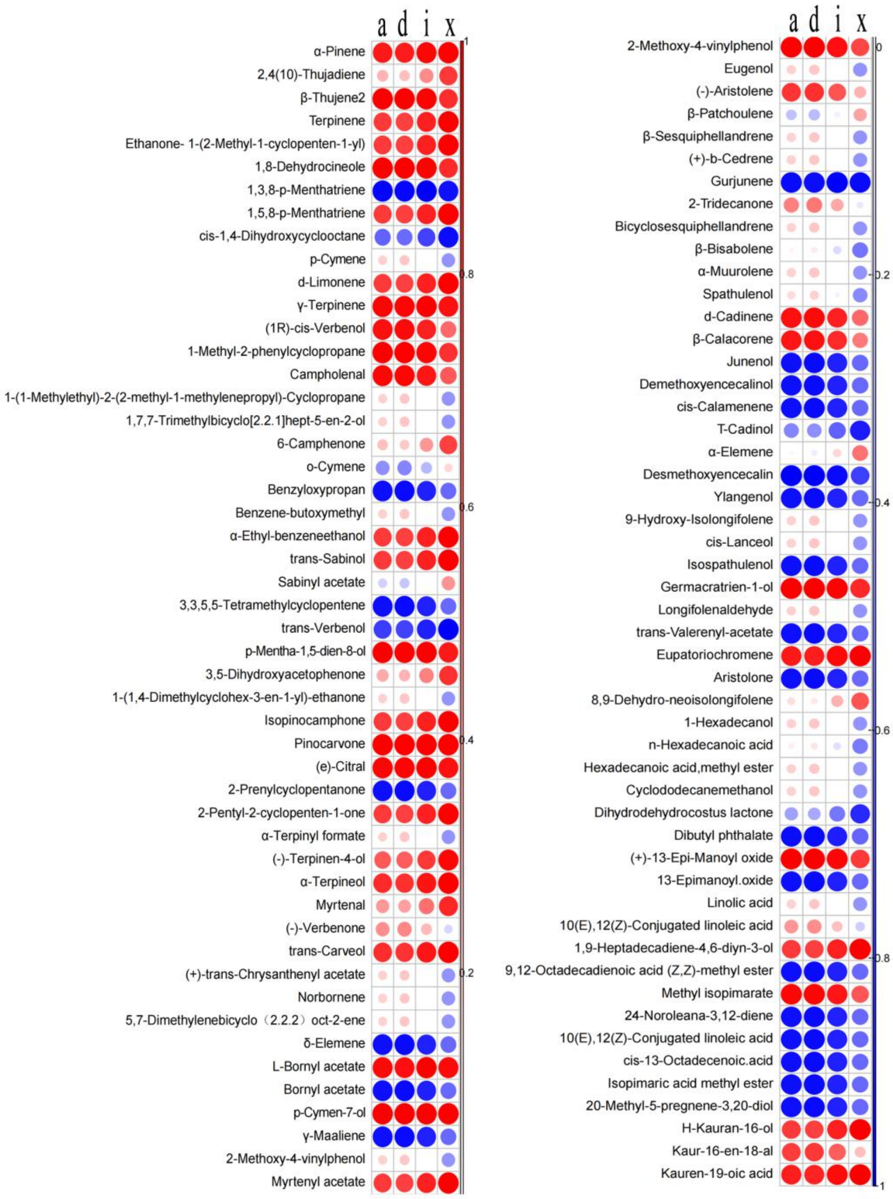

FIGURE S2 Correlation analysis of the chemical compounds of SEOs with antioxidant activities of SEOs and XO inhibitory activity. a means ABTS free radical scavenging ability, d means DPPH free radical scavenging ability, i means iron ion reduction ability, x means XO inhibition ability. Red circle means positive correlation, blue circle means negative correlation. The darker the color and the larger the circle, the higher the correlation between the relevant chemical compounds of SEOs and the biological activities.
